# Clinical outcomes of the PAUL Glaucoma implant for neovascular glaucoma

**DOI:** 10.1007/s00417-025-06933-3

**Published:** 2025-08-27

**Authors:** Carolin Deubel, Wolfgang Walz, Michael Petrak, Frank G. Holz, Raffael Liegl, Karl Mercieca, Constance Liegl née Weber

**Affiliations:** 1 Department of Ophthalmology, University of Bonn, Ernst-Abbe-Str. 2, D-53127 Bonn, Germany; 2 Department of Ophthalmology, Klinikum Dortmund, University Witten/Herdecke, Dortmund, 44145 Germany

**Keywords:** PAUL glaucoma implant, Secondary glaucoma, Neovascular glaucoma, Glaucoma surgery, Glaucoma drainage devices

## Abstract

**Purpose:**

In neovascular glaucoma (NVG), surgical interventions such as glaucoma drainage devices (GDD), may become necessary, especially when other therapies prove ineffective. The PAUL® Glaucoma Implant (PGI), with its refined, smaller drainage tube, presents a promising solution for lowering intraocular pressure (IOP) in such complex cases.

This study aims to evaluate the PGI’s effectiveness and safety in managing IOP in patients with NVG.

**Methods:**

This study reviewed medical records of patients who underwent PGI surgery for NVG at the University Hospital Bonn between May 2021 and January 2024. Preoperative and follow-up data, including IOP, BCVA, visual field progression, and complications, were collected prospectively. Success was defined using four IOP-based criteria (≤ 21, ≤ 18, ≤ 15, and ≤ 12 mmHg) per World Glaucoma Association (WGA) guidelines (World Glaucoma Association et al. 2009). Outcomes were classified as complete (without medication) or qualified (with or without medication). Failure included hypotony-related complications, need for further surgery, or PGI explantation. The primary endpoint was success rate by IOP criteria; secondary endpoints included changes in IOP, BCVA, medication use, complications, and impact of Prolene stent removal.

**Results:**

The study analyzed 23 eyes from 22 patients undergoing PGI surgery, with the majority being male (60.9%) and Caucasian (95.7%), and with an average age of 65.2 years. Success rates were highest for IOP ≤ 21 mmHg (60%–80% at 12 months) and declined with stricter thresholds, with only 10% maintaining IOP ≤ 12 mmHg. Prior to surgery, the mean intraocular pressure (IOP) was 27.22 mmHg. Following the procedure, a substantial reduction was observed, with IOP decreasing to 12.95 mmHg at the 12-month follow-up—representing an average decrease of 53.1%.

**Conclusion:**

The PGI demonstrates significant IOP reduction in NVG, with sustained success at higher IOP thresholds. However, maintaining very low IOP levels remains challenging.

## Key messages

*****What is known***:**

- Glaucoma drainage devices (GDDs) are widely used in neovascular glaucoma (NVG) due to limited success with filtering or cyclodestructive procedures.
- The PAUL® Glaucoma Implant (PGI) has shown promising results in various refractory glaucomas but had not been studied specifically in NVG patients.

*****What is new***:**

- The PGI significantly reduced intraocular pressure (IOP) in patients with neovascular glaucoma, achieving a 53.1% IOP reduction at 12 months—from 27.22 mmHg preoperatively to 12.95 mmHg.
- Kaplan–Meier analysis showed high success rates at moderate IOP thresholds (60–80% at IOP ≤ 21 mmHg), with fewer patients reaching stricter targets (only 10% for IOP ≤ 12 mmHg).
- PGI outcomes in NVG were comparable or superior to Ahmed and Baerveldt implants, with a manageable complication profile and no vision-threatening adverse events.

## Introduction

Neovascular glaucoma (NVG) is a serious complication of ocular ischemia caused by conditions such as retinal vein occlusion, diabetic retinopathy, and ocular ischemic syndrome. NVG poses a challenge for clinicians due to its high complexity. Despite advancements in anti-vascular endothelial growth factor (VEGF) therapy, blindness remains common in NVG due to anterior chamber angle obstruction by rubeosis and fibrovascular proliferation, which blocks aqueous humor outflow [2–4]. As for other types of glaucoma, topical and laser therapies are the first-line approach to lowering intraocular pressure (IOP). However, in advanced or therapy-resistant cases, surgical intervention becomes necessary [5]. Surgical options for NVG include filtering surgery with 5-fluorouracil or mitomycin C (MMC), glaucoma drainage device (GDD) implantation, and cyclodestructive procedures. Due to the limited success of filtering and cyclodestructive procedures [6], GDD implantation has become a preferred approach, particularly for patients unresponsive to medication; it has proven to be particularly effective in the management of secondary glaucoma [7–12]. However, meta-analyses on NVG surgery highlight the lack of high-quality studies, and no randomized controlled trials have yet compared surgical procedures for NVG, making consensus on optimal management challenging [13, 14].

The PAUL® Glaucoma Implant (PGI) is one of the newest GDDs and received CE marking in 2018. Distinguishing it from earlier devices such as the Baerveldt (BGI) and Ahmed (AGI) implants, the PGI features a notably smaller drainage tube (internal diameter: 0.127 mm, external diameter: 0.467 mm), offering a refined approach to controlling IOP [15]. Several studies have already demonstrated the PGI as a safe and effective intervention for reducing IOP in various forms of glaucoma such as uveitic glaucoma, secondary glaucoma following vitreoretinal surgery, and other refractory cases [16–18].

This study aims to assess the efficacy and safety of the PGI in patients with neovascular secondary glaucoma, focusing on its ability to reduce IOP and its safety profile in managing this complex condition. To our knowledge, no previous studies have evaluated the outcomes of the PGI in NVG.

## Materials and methods

### Participants

We performed a retrospective observational cohort study. The medical records of patients treated with PGI surgery for NVG at the Department of Ophthalmology, University Hospital Bonn, between May 2021 and January 2024 were reviewed. Data were collected prospectively starting from the time of surgery, with follow-up information gathered during each postoperative visit. The inclusion criteria were a confirmed diagnosis of neovascular glaucoma and a minimum follow-up duration of 12 months after PGI implantation. The exclusion criteria were the presence of any other type of glaucoma or insufficient follow-up duration. Comorbid systemic or ocular conditions such as diabetes mellitus, retinal vein occlusion, or prior interventions—including laser treatments, intravitreal injections, or previous ocular surgeries—were not considered exclusion criteria, as these factors are commonly associated with NVG and reflect the real-world complexity of this patient population.

All patients underwent a thorough ophthalmic examination upon presentation, including best-corrected visual acuity (BCVA) measurement using the Snellen chart (converted to logMAR for statistical analysis), Goldmann applanation tonometry for IOP, slit-lamp biomicroscopy, fundus examination, and visual field testing using the Humphrey 24–2 strategy (Carl Zeiss Meditec, Inc., Dublin, CA). For each patient undergoing PGI implantation, preoperative data such as gender, age, glaucoma type, BCVA, IOP, signs of anterior segment pathology, and specific clinical features were documented. Follow-up data included BCVA, IOP levels, visual field progression, complication rates, glaucoma medication usage, and details of further laser treatment, intravitreal injections (IVI) or surgeries. To assess success, four IOP-based criteria were applied according to WGA guidelines [1]: 1) Criterion-A: IOP ≤ 21 mmHg; 2) Criterion-B: IOP ≤ 18 mmHg; 3) Criterion C: IOP ≤ 15 mmHg; 4) Criterion-D: IOP ≤ 12 mmHg. A successful outcome was classified as complete if IOP was controlled without the need for glaucoma medication and as qualified if IOP control was achieved either with or without medication. Failure was was defined as any of the following: a single occurrence of IOP outside the predefined target range (IOP > 21 mmHg or < 6 mmHg) at any visit beyond three months postoperatively, the development of complications such as persistent IOP below 6 mmHg with hypotony-related issues, the requirement for further glaucoma surgery due to elevated IOP or explantation of the PGI device. This definition was applied consistently throughout the 12-month follow-up period. The primary endpoint of the study was the success rate based on the aforementioned IOP criteria, while secondary outcomes focused on changes in IOP, BCVA, use of IOP-lowering medications, complication rates, and the effects of Prolene intraluminal stent removal. All data were analyzed anonymously using de-identified information.

### Surgical method

The PGI is a novel drainage device, its plate is crafted from medical-grade silicone. Silicone offers a soft, large surface area for effective aqueous absorption while remaining flexible under the rectus muscles [19].

In most cases, the PGI is positioned in the superotemporal quadrant, alternative placements in the superonasal or inferonasal quadrants can be employed when necessary.

Surgical exposure is achieved through a conjunctival and Tenon’s peritomy in the chosen quadrant. Mitomycin-C (MMC), at a concentration of 0.5 mg/mL, is applied for two minutes to the area designated for the implant plate. The PGI plate is then inserted beneath the rectus muscles and secured to the sclera using 9–0 nylon sutures approximately 10 mm posterior to the corneal limbus. A 6–0 polypropylene stent is inserted into the tube to restrict flow. A 26-gauge cannula is used to create a tunnel into the anterior chamber. After trimming the tube to the required length, it is inserted through the tunnel into the anterior chamber and anchored using a 9–0 Nylon suture in a box configuration. To cover and protect the tube, a double-layered pericardial or fascia lata patch graft is applied with the aid of fibrin glue. The Prolene stent is typically tucked into a subconjunctival pocket in the inferior quadrant. The procedure concludes with conjunctival closure using two 10–0 Nylon slip-knot sutures at the corners and two additional 10–0 Nylon mattress sutures [15]. Postoperatively, the polypropylene stent can be removed once IOP exceeds the target level. Although there is no predefined removal timeline, stent extraction is generally deferred until at least eight weeks after surgery.

### Ethics approval

Ethics approval was granted by the local Ethics Committee at the University Hospital Bonn, and the study protocol followed the ethical standards outlined in the Declaration of Helsinki (2000), with prior approval from the Human Research Committee of the institution.

### Statistical analysis

Statistical analysis was conducted using SPSS Statistics version 27.0.0 (IBM Corporation, New York). The Kaplan–Meier method was used to estimate survival probabilities over time. Survival durations were calculated in 95% confidence intervals (CIs). The paired sample t-test was used to test differences between each pair of time points.

## Results

The demographic and clinical characteristics of 23 eyes from 22 patients undergoing PGI surgery are summarized in Table 1. The study population was mostly male (60.9%). The average age was 65.22 years, ranging from 38 to 84 years.

**Table 1:** Demographics and clinical characteristics of patients undergoing PGI surgery

	*n* = 23 eyes, 22 patients
Gender	
Male/female	14 (60.9)/9 (39.1)
Ethnicity	
Caucasian	22 (95.7)
Arab	1 (4.3)
Age	
Mean (± SD)	65.22 (± 11.52)
Range	38–84
Previous glaucoma surgery	
Yes	9 (39.1)
Which surgery	
Canaloplasty	1 (4.3)
Trabectome	3 (13.0)
Kahook Blade	1 (4.3)
CPC	6 (26.1)
Duration between initial diagnosis of NVG and PGI Implantation (years)	
Mean (± SD)	1.7 (± 1.67)
Range	0–5
Diagnosis of neovascular glaucoma	
Central retinal vein occlusion (CRVO)	8 (34.8)
Proliferative diabetic retinopathy (PDR)	12 (52.2)
Combined (CRVO + PDR)	1 (4.3)
Ocular ischaemic syndrome	2 (8.6)
Anesthesia	
Local	1 (4.3)
General	22 (95.7)
Number (#) of glaucoma drops	
Mean (± SD)	3.61 (± 1.03)
Range	1–4
Acetazolamide	
Yes	15 (65.2)
Lens status	
Phakic. Pseudophakic;	5 (21.7)/18 (78.37)
BCVA preoperatively (logMAR)	
Mean (± SD)	1.2 (± 0.77)
Range	0.2–2.7
IOP preoperatively	
Mean (± SD)	27.22 (± 9.33)
Range	10—44
Maximum IOP preoperatively	
Mean (± SD)	38.23 (± 11.15)
Range	26–72
Mean Deviation (MD) (Humphrey 24–2)	
Mean (± SD)	−19.96 (± 7.18)
Range	−10—−30
Pattern Standard Deviation (PSD)	
Mean (± SD)	8.71 (± 2.35)
Range	5.61—11.95

In terms of prior glaucoma surgeries, 39.1% of the patients had undergone previous procedures, most commonly transscleral cyclophotocoagulation (CPC) (26.1%).

The most common causes for NVG were proliferative diabetic retinopathy (PDR) (52.2%) and central retinal vein occlusion (CRVO) (34.8%).

Most eyes were pseudophakic (78.3%). Preoperative IOP had a mean value of 27.22 mmHg, ranging from 10 to 44 mmHg, with a maximum IOP reaching an average of 38.23 mmHg (range 26 to 72 mmHg). Patients took an average of 3.61 glaucoma drops [1, 2,3,4 preoperatively with 65.2% on acetazolamide. Mean preoperative BCVA was1.2 logMAR, ranging from 0.2 to 2.7.

Patient characteristics regarding NVG are shown in Table 2. On average, patients had undergone 1.5 previous vitreoretinal surgeries.

**Table 2:** Details regarding neovascular glaucoma

Amount of previous vitreoretinal surgeries	
Mean (± SD) (Range)	1.5 (± 0,79) (1—2)
1	4 (17.4)
2	4 (17.4)
IVI therapy	
Yes	20 (87)
No	3 (13)
What kind of IVI therapy	
Bevacizumab	17 (73.9)
Aflibercept	2 (8.7)
Ranibizumab	1 (4.3)
Dexamethasone	2 (8.7)
Amount of IVI therapy	
Mean (Range)	9 (1—32)
Diabetes mellitus	
Yes	16 (69.6)
No	7 (30.4)
Panretinal laser coagulation	
Yes	18 (78.3)
No	5 (21.7)

Regarding IVI therapy, 87% of patients had received it prior to surgery, with bevacizumab being the most common medication used (73.9%). Pan-retinal laser photocoagulation had been performed on 78.3% of patients. In terms of comorbidities, 69.6% of patients had diabetes mellitus, with a mean HbA1c level was 7.2%, (range 6.5 to 9.0).

**Fig. 1:**
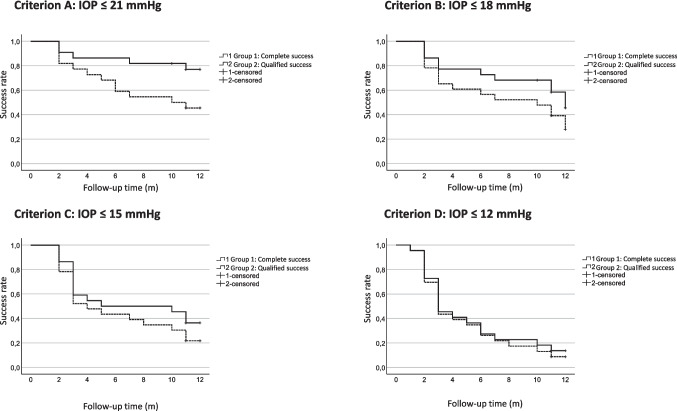
The Kaplan–Meier survival curves (Fig. 2) illustrate the probability of maintaining intraocular pressure (IOP) below specific thresholds over a 12-month follow-up period. Each graph represents a distinct success criterion (95% CI), with separate curves for complete success (Group 1), qualified success (Group 2). At 12 months, success rates exceed 60% for Criterion A (IOP ≤ 21 mmHg) but decline with stricter thresholds. For Criterion B (IOP ≤ 18 mmHg), complete success falls to around 40%, with qualified success at 30%. At IOP ≤ 15 mmHg (Criterion C), both rates drop below 30%, with complete success nearing 20%. The most stringent criterion D (IOP ≤ 12 mmHg) shows the lowest success, with rates around 10%

Primary outcomes were related to the above-mentioned success criteria. Qualified and complete success rates (95% CI) were 78% and 47% for Criterion A (IOP** ≤ **21 mmHg), 48% and 28% for Criterion B (IOP ≤ 18 mmHg), 38% and 22% for Criterion C (IOP ≤ 15 mmHg) and 15% and 10% for Criterion D (IOP ≤ 12 mmHg) (Fig. 1). The Kaplan–Meier survival analysis shown in Fig. 1 was used to assess surgical success based on predefined abovementioned IOP thresholds. Four criteria were evaluated over a 12-month follow-up period, each corresponding to a different target IOP level. Success rates were stratified into complete success (without medication) and qualified success (with medication). For the least stringent threshold (Criterion A: IOP ≤ 21 mmHg), the complete success rate exceeded 45%. With increasing stringency, success rates declined.

Table 3 presents the IOP and its percentage reduction at various time points post-surgery. Before surgery, the average IOP was 27.22 mmHg (range 10–44 mmHg). By Month 3, IOP slightly increased to 14.26 mmHg (range 7–22 mmHg) (p < 0.001), with a 42.29% reduction (range 0% to 75%). At Month 6, the average IOP was 12.27 mmHg (range 0–22 mmHg), showing a 50.89% reduction (range 0% to 100%)(p < 0.001). By Month 12, the average IOP was 12.95 mmHg (range 8–19 mmHg) (*p* < 0.001), reflecting a 53.11% reduction (range 0% to 100%).

**Table 3:** IOP and percentage reduction

Time point	IOP (range)	Percentage reduction
*Maximum IOP*	38.23 (26–72)	-
*Preoperative*	27.22 (10–44)	-
*Day 1*	11.96 (6–24)	50.18 (0–81.25)
*Month 1*	11.39 (6–25)	51.91 (0–84.61)
*Month 3*	14.26 (7–22)	42.29 (0–75.0)
*Month 6*	12.27 (0–22)	50.89 (0–100)
*Month 12*	12.95 (8–19)	53.11 (0–100)

**Fig. 2:**
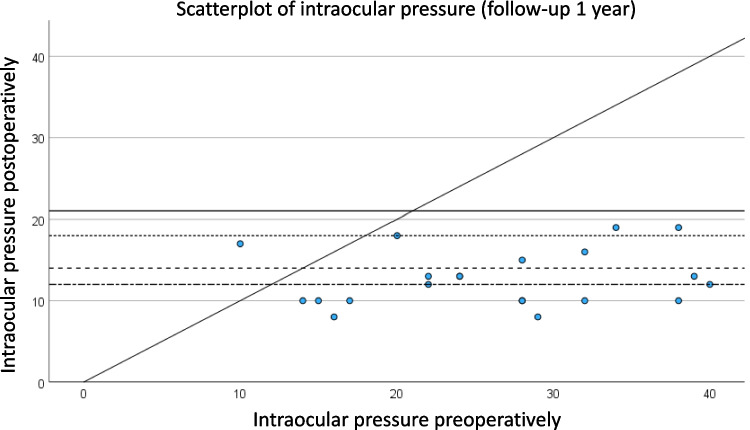
The scatterplot (Fig. 1) represents the relationship between preoperative and postoperative intraocular pressure (IOP) one year after surgery. The x-axis indicates the preoperative IOP, while the y-axis shows the IOP measured one year after the procedure. Each point represents an individual case, illustrating how the pressure changed over time

The relationship between preoperative and postoperative IOP one year after surgery is visualized in a scatterplot (Fig. 2), where each data point corresponds to an individual case. The plot demonstrates the degree of IOP reduction across the cohort, with the x-axis representing preoperative IOP values and the y-axis indicating IOP measured 12 months postoperatively. The distribution of points illustrates a consistent trend toward lower postoperative pressures, highlighting the overall effectiveness of the procedure in reducing IOP.

Table 4 shows complications following PGI surgery. Overall, 39.1% of patients experienced complications. No intraoperative complications were reported. Within the first three months, 4 eyes (17.4%) experienced hypotony with choroidal detachment, one of whom exhibited this shortly after Prolene removal. 2 patients (8.6%) required tube revision due to conjunctival dehiscence.

**Table 4:** Complications of PGI surgery

Time Point	Complications	Number of pat. (%)
Intraoperative	None	
First postoperative week	Hyphema	2 (8.6)
	Hypotony with choroidal detachment	2 (8.6)
First three postoperative months	Hypotony with choroidal detachment	2 (8.6)
	Conjunctival dehiscence	2 (8.6)
After the first three postoperative months	Hypotony with choroidal detachment	1 (4.3)
	Tube Revision due to conjunctival dehiscence	2 (8.6)

Several postoperative interventions were required in the months following PGI surgery to manage both glaucoma-related and unrelated complications. At 6 months post-surgery, one patient (4.3%) required an injection of viscoleastic into the anterior chamber to manage hypotony. Another patient (4.3%) underwent iridozonulohyaloidectomy followed by anterior vitrectomy and tube ligation due to aqueous misdirection, followed by cyclophotocoagulation one month later. Two patients (8.6%) needed revision of the PGI between 5 and 16 months after surgery due to conjunctival dehiscence and erosion. Additionally, one patient (4.3%) had the PGI explanted due to recurrent conjunctival erosions despite revision surgery and replaced with an Ahmed valve 11 months after surgery.

In terms of non-glaucoma-related procedures, 21.7% of patients continued receiving IVI after PGI surgery. Other procedures included vitrectomy due to vitreous hemorrhage due to retinal vein thrombosis which was performed six months after PGI surgery (4.3%), silicone oil removal (4.3%), and pan-retinal laser photocoagulation (8.6%). These additional treatments were performed to address complications or conditions related to neovascular glaucoma and did not affect the IOP or lead to further complications.

An intraluminal prolene stent was removed in 12 eyes (52.2%) after a mean time period of 5.23 months (1–17 m). Mean IOP before the removal was 27.25 mmHg (19–38 mmHg) and decreased to 11.75 mmHg (3–22 mmHg).

## Discussion

GDD are commonly used as a first-line treatment for NVG. The preference for tube shunts as an initial treatment option in NVG was demonstrated in a study by Iwasaki et al. [20], among others. However, meta-analyses indicate that the lack of high-quality studies prevents a clear consensus on NVG management, as no randomized trials compare different surgical procedures [13]. A meta-analysis of seven non-randomized studies by Schomak et al. [21] suggests that while GDD and CPC show similar efficacy, GDD are safer [21]. Multiple studies report significant IOP reduction following GDD implantation in NVG, supporting its role as a viable surgical option [22].

The PGI is a novel device that is already frequently used in therapy-refractory glaucoma [8, 16, 18]. However, to date, no study has provided insights into the outcomes of the PGI in NVG. Our study results demonstrated a significant and sustained IOP reduction following PGI implantation for NVG patients.

In our study, the IOP decreased from 27.22 mmHg preoperatively to 12.95 mmHg after 12 months– a reduction of over 50%., indicating that PGI provides an effective medium-term solution for IOP control in therapy-resistant NVG cases. This outcome is notably favorable when compared with the Ahmed Glaucoma Valve (AGV) study by Netland et al., where mean postoperative IOP was 16.5 ± 15.8 mmHg at one year. [23]. Although our cohort achieved lower IOP, it is important to consider that the Netland study included eyes with a higher baseline IOP (43.8 ± 11.0 mmHg), which may have impacted their final values. Moreover, our frequent use of acetazolamide preoperatively might have contributed to a more stabilized IOP profile postoperatively, a factor not detailed in their study. Similarly, Noor et al. observed an IOP of 12.0 mmHg after 9 months in their study (AGV and BGI) in the group that received bevacizumab, which aligns closely with our results [24]. This suggests that the combination of anti-VEGF therapy with drainage devices may play a key role in optimizing IOP control in NVG, however, we did not use any anti-VEGF medication in our study. Iwasaki et al. [25] conducted a large multicenter retrospective study comparing BGI and AGV in NVG, [25] reported similar IOP values one year postoperatively, with 14.1 ± 5.2 mmHg in the BGI group and 13.8 ± 4.2 mmHg in the AGV group. Sidoti et al. [22] and Nishitsuka et al. [26] also reported significant IOP reductions using the BGI, with postoperative IOPs of 11.2 ± 3.9 mmHg and 14.8 mmHg at 12 months, respectively. In another multicenter study, Iwasaki et al. [20] compared BGI and trabeculectomy in NVG and found that although trabeculectomy yielded a lower IOP at 1 month (14.3 ± 7.2 mmHg vs. 18.8 ± 9.7 mmHg, *P* < 0.01), by 12 months the IOPs were nearly identical between the two groups (13.8 ± 6.1 mmHg for BGI vs. 13.9 ± 5.6 mmHg for trabeculectomy), reinforcing the long-term effectiveness of tube surgery. Our outcomes are therefore well within the range reported in these established BGI studies. While the absolute IOP reduction in our cohort may appear less pronounced than in some earlier reports, this is partly attributable to the lower baseline IOP in our patients. Nevertheless, the final IOP levels achieved remain clinically meaningful and demonstrate the PGI’s effectiveness in achieving target pressure even in less extreme preoperative cases.

The longer-term outcomes, such as the three-year results from Maeda et al., also support the use of large-plate GDDs in NVG, with IOP decreasing from 34.8 ± 9.1 mmHg to 15.6 ± 4.6 mmHg in the AGV group, and from 36.9 ± 9.2 mmHg to 12.8 ± 5.5 mmHg in the BGI group [27]. Our 12-month IOP data are comparable to or even slightly lower than those three-year values, indicating that the PGI may be similarly effective in the medium term. However, longer follow-up is needed to confirm whether PGI can maintain such control over time. Additionally, Shalaby et al. [28] reported similar failure rates between AGI and BGI devices (21.6% vs. 25.9%; *P* = 0.552), suggesting no clear advantage between these two traditional implants in terms of long-term outcomes—a comparison that further supports the PGI as a viable alternative. In their study, the mean IOP dropped after 6 months significantly in both groups: from 41.4 to 16.5 mmHg in AGV eyes and from 39.3 to 16.5 mmHg in BGI eyes (both *P* < 0.0001).

In terms of success rates, our findings were slightly lower than those reported by Shen et al. [29], who noted success rates of 70% and 65% for trabeculectomy and the Ahmed valve, respectively, versus approximately 60% and 50% in our study for the respective success criterion. However it has to be considered that success rate definitions vary between studies and are not directly comparable. Compared with Noor et al. [24], our results were more consistent with their bevacizumab-treated group (87% success compared to around 53% for the group without), though differences in the timing of injections make direct comparison difficult. Sidoti et al.’s BGI study [22] reported a one-year success rate of 79%, and Nishitsuka et al. [26] reported 100% at one year, but these studies used differing definitions of success and included broader patient populations. Iwasaki et al. [25] found that BGI yielded significantly higher success rates than AGV for IOP targets of < 21 mmHg and < 17 mmHg, with a lower re-operation rate and comparable complication profiles. In a separate study, Iwasaki et al. [20] also reported significantly higher success rates for BGI compared to trabeculectomy at one year in NVG patients (*P* < 0.01 for IOP > 21 mmHg; *P* = 0.01 for IOP > 17 mmHg), along with a lower reoperation rate (5.0% vs. 20.1%). When viewed in context, our success rates remain clinically meaningful given the NVG-specific focus and real-world complexity of our cohort.

Larger GDD are generally associated with greater IOP reduction, presumably due to the increased surface area available for aqueous humor filtration. The PGI stands out among GDDs due to its unique tube dimensions—featuring an internal diameter of 0.127 mm and an external diameter of 0.467 mm—compared to the BGI and AGV, which typically have tube diameters of 0.305/0.635 mm and 0.30/0.63 mm, respectively. Despite the PGI’s smaller tube caliber, its end-plate surface area (342 mm^2^) is comparable to those of the traditional large-plate devices, such as the BGI (available in 250 mm^2^ and 350 mm^2^ models) and the AGV (ranging from 102 mm^2^ to 364 mm^2^, depending on the model). Despite similar plate sizes and smaller tube lumen, our results for the PGI are similar or even better than in the published literature. This may be explained by several factors, including the material (medical-grade silicone), biocompatibility and flexibility of the plate, which potentially allows for a more smooth and diffuse flow within the capsule. These characteristics may also contribute to a potentially improved safety profile, with fewer complications related to mechanical irritation or fibrosis. However, while short- and medium-term results are encouraging, current evidence on the PGI is limited to a maximum follow-up of three years [16, 30]. It remains unclear whether the favorable IOP outcomes can be maintained over longer durations, particularly when compared to the more extensively studied BGI and AGV. Ongoing long-term studies will be essential to determine the durability of PGI performance and to validate its role in the long-term management of refractory glaucoma. In our clinical practice, the PGI has already become the standard implant for neovascular glaucoma in eyes with visual potential. Its combination of effective IOP control and lower risk of hypotony has made it the preferred choice over other devices.

To date, no studies have been published specifically examining the outcomes of the PGI in patients with NVG, making direct comparisons challenging. Nonetheless, when comparing our NVG-specific results with the broader PGI literature, our postoperative IOP values are notably similar to those reported across diverse glaucoma populations. For instance, we observed a mean IOP of 12.95 mmHg at 12 months, which closely aligns with mean IOPs reported in previous PGI studies: 13.2 mmHg (Koh et al. [31]), 13.3 mmHg (Vallabh et al. [32]), 12.5 mmHg (José et al. [17]), 14.2 mmHg (Tan et al. [30]), and 12.07 mmHg (Weber et al. [33]). These parallels suggest that the PGI may be capable of achieving effective IOP control in NVG, a particularly aggressive and refractory subtype of glaucoma, to a degree comparable with its performance in more common glaucoma types. However, when examining success rates, a divergence emerges. Studies by Weber et al. and Koh et al. reported higher one-year qualified success rates—95.6% and 93.2%, respectively—whereas our study observed lower success rates of approximately 60%. This difference likely reflects the inherent challenges associated with treating NVG, which is characterized by rapid disease progression, poor visual prognosis, and a higher incidence of postoperative complications. Additionally, Vallabh et al. reported success rates of 91.1% and 38.4%, but their cohort included a heterogeneous glaucoma population, further complicating direct comparison.

Taken together, our findings suggest that while the PGI can achieve IOP reductions in NVG comparable to those seen in other glaucoma types, success rates may be lower due to the more severe underlying pathology. These results support the PGI as a viable surgical option in NVG, while also emphasizing the need for future studies focused specifically on this high-risk patient group to better define long-term outcomes and refine success criteria.

The PGI is thought to reduce postoperative complications, particularly hypotony, due to its smaller inner lumen diameter. Despite the promising IOP reduction observed, our study also revealed several complications associated with PGI surgery, though none were vision-threatening. Overall, 39.1% of patients experienced some form of complication, with hypotony-related issues, particularly choroidal detachment, being the most common (8.7% in the first week and 8.6% within three months). This is not a rare complication and has also been reported as a complication in other studies like from Weber et al. [33] (hypotony rate 8.9% in the first 3 months postoperatively) or Tan et al. [30] (hypotony rate 35.4%), although our study showed slightly higher hypotony rates compared to the study from José [17] (hypotony rate 2.8%). This may be attributed to our smaller sample size and the inclusion of only NVG patients, who generally have higher complication rates.

Additionally, some patients required intervention for conjunctival erosion, with a few needing tube revision or explantation of the PGI device. These complications are not uncommon in NVG cases, given the multifactorial etiology of the condition, including new vessel formation and ongoing inflammation [4].

In patients with NVG, one would typically expect a higher rate of hyphema due to the presence of neovascularization in the angle. The newly formed blood vessels in NVG are fragile and prone to rupture, making hyphema more common. These fragile vessels are more susceptible to trauma during surgical intervention, which could lead to postoperative bleeding. However, in our study, the rate of hyphema was relatively low, with only 8.7% of patients experiencing this complication in the first week after surgery. This finding is noteworthy, as it differs from what might have been anticipated given the degree of neovascularization in these patients. However, when compared to the BVI studies by Sidoti et al. [22] or Nishitsuka et al. [26], a similar rate of hyphema can be observed (8% and 14.80%). From this, it can be concluded that despite the smaller size of the PAUL tube, the complication rate related to hyphema remains comparable.

The need for non-additional glaucoma-related procedures, including vitrectomy or further IVI, highlights the complexity of managing NVG, particularly when secondary complications occur. One patient underwent silicone oil removal after PGI surgery. Due to the urgent need for IOP control and the VR surgeon’s wish to keep the silicone oil in situ longer, we prioritized PGI implantation first to stabilize IOP. Silicone oil removal was subsequently performed once the retinal condition had stabilized. In similar situations, we may choose a staged approach—implanting the PGI first when urgent IOP control is needed and the silicone oil is intended to remain in place for a longer period. In our experience, the PGI has also proven to be effective in eyes where the silicone oil remains permanently in situ for retinal reasons [8]. Further, in selected cases we perform combined vitrectomy (± silicone oil removal or -exchange) and PGI implantation, when both pressure control and VR intervention are simultaneously required, this approach remains the exception rather than the rule in our clinical practice. These non-glaucoma related procedures did not affect the IOP after PGI implantation. While the PGI is effective in reducing IOP, it emphasizes the importance of careful monitoring and, if necessary, further interventions to manage complications and ensure long-term success.

Our study provides valuable demographic insights, showing that the majority of patients had advanced glaucoma with extensive prior treatments, including multiple vitreoretinal surgeries and IVI. The high prevalence of comorbidities, such as diabetes mellitus (69.6%), underscores the chronic and multifactorial nature of NVG, which complicates both treatment and outcomes. The severity of the disease is further reflected in the mean preoperative visual acuity (logMAR 1.2) and intraocular pressure (IOP) (mean 27.22 mmHg), indicating poor visual prognosis before surgery. Despite the advanced stage at presentation, our findings suggest that vision was preserved postoperatively, reinforcing the value of a more invasive GDD over CPC in these high-risk cases. By effectively reducing IOP and stabilizing the disease, PGI offers a crucial opportunity to maintain functional vision, which may justify its surgical invasiveness. Additionally, the reduction in the need for topical medications postoperatively may contribute to an improved quality of life for patients, alleviating both the financial and physical burdens of long-term drop therapy. Given the urgency of preserving any remaining visual function, a safe and effective therapeutic approach is essential. PGI presents a promising option, particularly for patients in whom other treatments have failed or proven inadequate, helping to prevent further optic nerve damage and maintain residual vision.

The aforementioned studies confirm that patients with NVG often present with poor initial visual acuity, posing ongoing challenges for ophthalmology. For instance, in the study by Medert et al. [34], preoperative visual acuity was already very low, ranging from 1.45 to 1.7 logMAR, with slightly better outcomes observed in patients with proliferative diabetic retinopathy (PDR) compared to those with retinal vein occlusion (RVO). This highlights the critical importance of early intervention in neovascular glaucoma to optimize visual outcomes.

### Limitations

One limitation of this study is the relatively small sample size (23 eyes from 22 patients), which may limit generalizability of the findings. Due to this, it was not possible to compare different subtypes of neovascular glaucoma, such as diabetic neovascular glaucoma versus veno-occlusive neovascular glaucoma, or draw meaningful conclusions regarding their distinct outcomes. Additionally, the follow-up period of 12 months, while informative, may not fully capture the long-term efficacy and complications associated with PGI, especially given the chronic nature of NVG. Another limitation is the study's predominantly Caucasian population (95.7%), which may limit the applicability of the results to more ethnically diverse groups. Furthermore, the study does not include assessments of patient-reported outcomes or quality of life, which are crucial for evaluating the overall success of glaucoma treatments from the patient's perspective. Lastly, endothelial cell counts were not routinely measured in this cohort, which limits our ability to assess the potential impact of the PGI on corneal endothelial health. Addressing these aspects in future research would provide a more comprehensive understanding of the PGI's impact on both clinical outcomes and patient well-being.

## Conclusion

In summary, the treatment of NVG typically involves a comprehensive therapeutic approach, including panretinal photocoagulation, anti-VEGF therapy, and IOP-lowering treatments [35–37].The PGI demonstrates substantial effectiveness in lowering IOP in patients with neovascular secondary glaucoma, offering a reliable solution for those with refractory cases. However, the study also highlights the challenges of maintaining low IOP levels over time. The observed complications and the need for additional interventions suggest that while the PGI is a valuable tool, its application requires careful patient selection, close monitoring, and potentially adjunctive treatment strategies to optimize long-term outcomes.

In conclusion, the PGI is an effective and reliable solution for managing neovascular glaucoma, offering significant intraocular pressure reduction and providing a valuable treatment option for refractory cases.

## Data Availability

All datasets generated during and/or analyzed during the current study are available from the corresponding author on reasonable request. The data that support the findings of this study are not publicly available because they contain information that could compromise the privacy of research participants, but are available from CW upon reasonable request.
